# Enhancing recovery of bioactive compounds from *Cosmos caudatus* leaves via ultrasonic extraction

**DOI:** 10.1038/s41598-021-96623-x

**Published:** 2021-08-27

**Authors:** Norliza Abdul Latiff, Pei Ying Ong, Siti Nor Azlina Abd Rashid, Luqman Chuah Abdullah, Nor Amaiza Mohd Amin, Noor Akhmazillah Mohd Fauzi

**Affiliations:** 1 Department of Chemical and Environmental Engineering, Faculty of Engineering, Universiti Putra Malaysia, 43400 Serdang, Selangor Malaysia; 2 Department of Process and Food Engineering, Faculty of Engineering, Universiti Putra Malaysia, 43400 Serdang, Selangor Malaysia; 3 Innovation Centre in Agritechnology, Universiti Teknologi Malaysia, 84600 Muar, Johor Malaysia; 4 Department of Chemical Engineering Technology, Faculty of Engineering Technology, Universiti Tun Hussein Onn Malaysia, Pagoh Higher Education Hub, 84600 Muar, Johor Malaysia

**Keywords:** Energy science and technology, Engineering, Materials science

## Abstract

*Cosmos caudatus* (*C. caudatus*) is a medicinal plant that is high in bioactive compounds such as phenolics. In this study, an ultrasound extraction method was used to optimise the extraction of bioactive compounds from *C. caudatus* leaves. Response surface methodology (RSM) based on a Box-Behnken design (BBD) was applied to obtain the optimum extraction parameters which is solid–liquid ratio (10–30 g/mL), particle size (180–850 µm) and extraction time (20–30 min) for maximal quercitrin and total phenolic content (TPC) yields. Analysis of antimicrobial activity was performed against two human pathogenic microbes: *Staphylococcus aureus* (*S. aureus*) and *Escherichia coli* (*E. coli*) by the agar well diffusion method. The optimal ultrasonic extraction condition was as follow: solvent-liquid ratio of 1:28 (g/mL), particle size of 485 µm, and duration of 30 min, respectively. Remarkably, extraction using ultrasonic method had recovered more bioactive content and antioxidant activity than the Soxhlet method. The extract also exhibited good antimicrobial activities. Due to the above findings, the ultrasonic extraction was found to be suitable to improve recovery extraction of quercitrin and TPC from *C. caudatus* leaves. It also opens the possibility that the plant extract can be used for functional food and antimicrobial agents in various applications.

## Introduction

*Cosmos caudatus* (*C. caudatus*) is a small plant belonging to the Asteraceae family^1^. In Southeast Asia countries like Malaysia and Indonesia, the plant is usually consumed as food and used in traditional folk medicine^1,2^. The leaves and shoots can be eaten raw as a side dish with meals as well as for other culinary purposes^2,3^. Previous work has been done on the plant extraction mostly on the leaves which reported various pharmacological properties such as anti-bacterial, anti-inflammatory, bone-protective agent, and anti-diabetic^2,4–9^. Some studies have demonstrated that the extract has more powerful antioxidant properties contrasted to some tropical fruits, herbs, and vegetables^10,11^. In addition, the plant is also rich in vitamins (B1, B2, C and β-carotene), minerals (potassium, calcium, magnesium, phosphorus, iron, zinc, sodium and copper) and is low in calories^1,12^.

Phytochemical studies on this plant described various bioactive compounds such as quercetin derivatives (quercetin-3-*O*-rhamnoside, quercetin-3-rutinoside, quercetin glucoside), phenolics, ascorbic acid, proanthocyanins, and catechin^13–16^. Various researchers reported quercetin-3-*O*-rhamnoside or quercitrin as the major compound in the plant extract^9,14,16^. Previous studies also demonstrated that quercitrin possessed tremendous bioactivities such as antioxidant, antibacterial, osteoblast protection, and allergic prevention^17–20^. Thus, it is important to investigate the extraction and separation of bioactive compounds from the plant materials. To our best knowledge, research focusing on the extraction of specific bioactive compounds from the herb has not been yet reported. Therefore, in considering the beneficial prospects of the plant leaf extract as a source of antioxidant compounds from quercitrin, an efficient extraction method and process should be developed and proposed.

It is desirable to develop a strategy plant’s extraction method which can reduce the time and production costs yet applies the green concept with low energy and eco-friendly. Various modification methods with either ultrasonic, microwave, ultra-high pressure or supercritical fluid extractions assistance can be applied^21,22^. Ultrasound-assisted extraction, either using the ultrasonic bath or ultrasonic probe devices, could be the alternative and promising green extraction technology among the established methods^22–24^. It provides a faster, simpler operation method, has high reproducibility, and it is suitable for thermo-labile compounds^25–27^. The extraction efficiency can be increased by the acoustic cavitation in the solvent which leads to the disruption or breakdown of plant cell walls. This mechanism accelerates molecules movement and increases the mass transfer process in contact with solid material during the extraction process^28,29^. The parameters of ultrasonic extraction efficiency can be affected by ultrasonic irradiation time, temperature, frequency, the ratio between solvent to solid ratio, and solvent types^30,31^. Similarly, the applied extraction parameters should be carried out under optimal conditions to produce a high yield recovery of the targeted extraction. This can be achieved by carrying out a statistical design based on response surface methodology (RSM)^27^.

Studies conducted elsewhere have revealed various extraction methods for *C. caudatus*^32–35^, however, there are no reports about the recovery yield of the bioactive compound from *C. caudatus* leaves extract. Therefore, the present study utilised ultrasonic extraction to extract the bioactive compound and the parameter of extractions were then optimised using response surface methodology. The extract obtained under optimal ultrasonic extraction conditions were then compared to the Soxhlet extraction method in terms of yields and effectiveness. Furthermore, an analysis of antimicrobial activity was performed to evaluate the potential of plant extract as an antimicrobial agent against the following two human pathogens: *Staphylococcus aureus* (*S. aureus*) and *Escherichia coli* (*E. coli*).

## Materials and methods

### Plants sample collection and preparation of* Cosmos caudatus* extract

*Cosmos caudatus* (*C. caudatus*) was collected from the university’s research farm of Universiti Teknologi Malaysia, located at Pagoh, Johor (2.15582, 102.73446). The date of collection was in April 2019 and October 2019. The plant was authenticated by the botanist (Dr Mohd Firdaus Ismail), Institute of Bioscience, Universiti Putra Malaysia. After analysed, the plant specimens were deposited in the herbarium laboratory of Universiti Teknologi Malaysia, Pagoh. The fresh leaf parts were dried at 40 °C for 4 h in a laboratory oven dryer^12^. The dried leaves were subsequently ground into a powder and sieved (Wstyler, Mentor, OH, USA). The samples obtained were kept and sealed in an air-tight container and maintained at − 25 °C until further used.

### Chemicals and reagents

Quercitrin and gallic acid (3,4,5-trihydroxy benzoic acid, 99%) and Folin–Ciocalteu phenol reagent, Na_2_CO_3_, formic acid (HPLC grade), 1-diphenyl-2-picryl-hydrazyl (DPPH), and Streptomycin was procured from Sigma Aldrich (Steinheim, Germany). Ethanol (reagent grade) was purchased from Across Organics (Leicestershire, England). Methanol (HPLC grade) and Acetonitrile (HPLC grade) were obtained from Daejung Company, Ltd. (Busan, South-Korea).

### Ultrasonic extraction

Ultrasonic extraction was carried out in an ultrasonic bath device (i.e., WiseD; Daihan Scientific, Ltd Co, South-Korea) at a constant frequency of 40 kHz with 175 W of power. The ultrasonic time, temperature, and power intensity were controlled from the instrument panel. The dried and grounded samples were immersed into a 100 mL glass beaker containing a solvent (80% v/v ethanol)^13^. The beaker was put in the ultrasonic bath and the temperature was maintained at 50 °C. After completion of the extraction process, the samples were filtered into a vacuum pump and the clear supernatant was transferred into a concentrator centrifuge tube (Concentrator plus, Hamburg, Germany) and concentrated until dryness. Afterwards, the crude extract was refrigerated at − 25 °C to minimise the degradation of the bioactive compounds caused by oxidation, prior to subsequent analysis.

### Soxhlet extraction

Extraction was performed using the Soxhlet apparatus. The dried and grounder leaf sample was placed in a thimble chamber and inserted into the thimble holder condenser. It was attached to a 250 mL distillation flask. The sample (1 g) and 80% ethanol (200 mL) were used for the extraction process. The extraction was maintained for 5 h at 80 °C. After completing the extraction, the residue was filtered in a vacuum pump and concentrated until dryness using a solvent centrifugal concentrator (Concentrator plus, Hamburg, Germany). The samples were kept refrigerated at − 25 °C before analysis.

### High-performance liquid chromatography analysis (HPLC)

The chromatographic analysis of quercitrin from *C. caudatus* leaf extract was done using a 1290 Infinity HPLC coupled with a diode array detector system (HPLC–DAD) (Agilent Technologies, Santa Clara, CA, USA). A reverse-phase column (Inertsil ODS, 5 µm, 250 mm × 4.6 mm I.D.) was used for separation of the compound. The analysis of quercitrin was following Sharifuldin et al.^14^ with some adjustments. The injection volume was 10 µL. The elution set were 0.3% formic acid in water (A) and 100% acetonitrile (B) with the following sequence of A distributions as follows: 0 min-80%, 10 min-50%, 11 min-0%, 14 min-80%. The separation was achieved at a flow rate of 0.5 mL/min for a 14 min run time. The detection wavelength of quercitrin was achieved at 260 nm and the wavelength was integrated by OpenLab software (Agilent Technologies, Santa Clara, CA, USA). The quercitrin content was calculated using a linear regression equation that was obtained from the standard calibration curve of quercitrin and expressed as mg quercitrin per g of dry weight extract (mg/g dw). Before the injection, all samples were filtered using a nylon membrane 0.45 µm. The experiments were conducted in triplicate.

### Total phenolic content analysis by UV-spectrophotometric

The total phenolic content (TPC) of the leaf extract was analysed using the UV-spectrophotometric method by Safdar et al.^36^ with modifications. Briefly, a set of 40 µL extracts were added into a 96-well plate, followed by 100 µL Folin–Ciocalteau’s reagent (tenfold diluted). After 5 min of reaction, 80 µL of Na_2_CO_3_ (7.5 g/100 mL) was added to the sample solutions. The samples were shaken and incubated at ambient temperature (26 °C) to develop a blue colour. After 90 min, the samples were measured at an absorbance at 765 nm using an ELISA microplate reader (VersaMax, Molecular Devices, LLC, USA). The TPC was expressed as mg gallic acid equivalent per g of dry weight extract (mg GAE/g dw). All experiments were conducted in triplicate.

### Antioxidant analysis by UV-spectrophotometric

The antioxidant assay was determined using free radical scavenging method as referred to Latiff et al.^37^. Free radical agent, namely 1,1-diphenyl-2-picryl-hydrazyl (DPPH), was prepared in 100% CH_3_OH (reagent grade) with a concentration of 0.1 mM. A serial dilution of samples were inserted into a 96-well plate. The samples were mixed with 10 µL DPPH reagent. The mixtures were shaken in a platform shaker (Polymox 1040, Heidolph Instruments GmBH & CO. Schwabach, Germany) and allowed to stand at room temperature (26 °C), with minimal light exposure. After 30 min reaction, the samples absorbance was recorded at 515 nm using an ELISA microplate reader (VersaMax, Molecular Devices, LLC, USA). The ascorbic acid was used as a standard and DPPH (without extract) as a control sample was also prepared and similarly analysed. All experiments were conducted in triplicates. The reduction rate of DPPH was calculated with the following Eq. (1).

1 $$\mathrm{\% Inhibition}=\frac{{\mathrm{A}}_{s}-{A}_{s}}{{A}_{c}}\times 100,$$

where, A_c_ is the absorbance of the control, and A_s_ is the absorbance of extract.

### Antimicrobial analysis

Two clinical human pathogenic bacteria; *Staphylococcus aureus* (*S. aureus*) and *Escherichia coli* (*E. coli*) were used to determine the antimicrobial potential using the agar well diffusion method^38^. The nutrient agar was inoculated with bacteria and treated with the 50 µL plant extract (40 mg/mL). The same assay was also performed with standard compound of quercitrin (1 mg/mL). After the incubation period of 24 h under a controlled temperature (30 °C), the diameter of the inhibition zone was measured. Standard antibiotic of Streptomycin (10 mg/mL) was used as control in this study.

### Optimization of ultrasonic extraction by Box–Behnken experimental design

Prior to the study, a single-factor experiment was performed on four extraction variables [solid–liquid ratio (SLR), particle size, amplitude, and time] and its effect on the yield of quercitrin and TPC was analysed. The study was performed over 19 tests (with three replicates for each test) to determine its effect and range (see Supplementary Information). Based on the results of the single-factor experiments, parameters of SLR, particle size, and time were selected as the important parameters that significantly affect the quercitrin and TPC yields. These parameters have also been taken as the important for quercitrin and phenolic extraction in ultrasonic and other extraction process^39,40^. Table 1 showed the parameters used in the optimization study. Amplitude level was set constant at 40% in this study. A three-level Box–Behnken design (BBD) was performed to investigate and optimise the effect of the selected extraction variables. The mean values from the experimental works were fitted in a second-order polynomial equation to predict the responses using the Eq. (2).

**Table 1:** The selected independent variables and coded values.

Factors	Independent variables		
Levels	X_1_ (g/mL)	X_2_ (µm)	X_3_ (min)
− 1	10	180	20
0	20	515	25
+ 1	30	850	30

Parameters: X_1_ = solvent to solid ratio, X_2_ = particle size, and X_3_ = time.

2 $$Y={\beta }_{0}{\sum }_{\mathrm{i}=1}^{\mathrm{k}}{\beta }_{i}{x}_{i}+ {\sum }_{\mathrm{i}=1}^{\mathrm{k}}{\beta }_{ii}{{x}^{2}}_{i}+ {\sum }_{1\le \mathrm{i}\le \mathrm{j}}^{\mathrm{k}}{\beta }_{ij}{x}_{i}{x}_{j},$$

where, Y represents the predicted response (quercitrin and TPC), k stands for the number of factors determined (k=3) in this study, ${\beta }_{0},{\beta }_{i}, {\beta }_{ii,}{ \beta }_{ij}$ represent the coefficients term as linear, quadratic, and interactive, respectively, ${x}_{i,} {x}_{ii, }$ and ${x}_{ij}$ are the coded independent factors^22,41^.

The statistical data (*p*-value) among the independent and coefficient terms was evaluated by the analysis of variance (ANOVA). The data was generated using Minitab version 16 (Coventry, London). The result with small *p*-value (*p* ≤ 0.05) and larger F-value indicated that the model term was significant^42,43^. Contour and surface plots were also generated to visualize the interaction among the independent variables. The validity of the optimum conditions was checked through desirability values of the response (0 to 1), which value close to 1 shows the more optimal conditions^44,45^.

### Ethical statement

Research carried out as per guidelines and recommendations of “Malaysian Herbal Monographs 2015”, published by the Ministry of Agriculture and Food Industries of Malaysia. No permit or permission is required for collection of seeds and plants.

## Results and discussion

### Ultrasonic extraction parameter optimisation

In this study, the BBD was performed to determine the optimal independent variables for the maximal quercitrin and TPC yields from *C. caudatus* leaves. An extraction model based on a second-order polynomial equation was then developed. The results obtained from the actual works and the predicted values by the second-order polynomial equation model are shown in Table 2. The experimental values of quercitrin ranged from 20.81 to 54.11 mg/g dw, while the predicted values ranged from 21.05 to 55.45 mg/g dw. For the TPC, the experimental values ranged from 119.72 to 188.28 mg GAE/g dw, while the predicted values ranged from 118.05 to 188.78 mg GAE/g dw.

**Table 2:** BBD model fitness for optimum yield of quercitrin and TPC from *C. caudatus* leaves.

Run no.	X_1_	X_2_	X_3_	Quercitrin (mg/g dw)		TPC (mg GAE/g dw)	
				Experimental	Predicted	Experimental	Predicted
1	30	515	20	25.85	26.09	188.28	188.78
2	10	515	30	49.14	49.41	125.42	125.36
3	30	180	25	22.08	22.94	178.72	177.65
4	10	515	20	54.11	55.45	126.39	127.51
5	10	180	25	40.31	40.07	119.75	118.05
6	30	850	25	20.81	21.05	180.96	182.65
7	20	180	30	43.87	44.35	137.60	139.79
8	20	850	20	40.15	39.67	149.12	146.93
9	20	515	25	45.92	45.39	148.56	148.65
10	20	515	25	45.58	45.39	146.56	148.65
11	20	850	30	41.90	43.07	136.20	135.63
12	10	850	25	42.50	41.58	120.30	121.37
13	30	515	30	42.46	41.11	186.09	184.97
14	20	180	20	38.98	38.71	133.88	134.45
15	20	515	25	44.67	45.39	150.82	148.65

X_1_ = solvent to solid ratio, X_2_ = particle size, and X_3_ = time.

The ANOVA data of the predicted models is displayed in Table 3. In this study, the model was significant for the two respective responses. The presented data showed the best fit with higher R-square value (R^2^) corresponding to 0.9930 for quercitrin and 0.9962 for TPC. The adjusted R^2^ value for quercitrin was 0.8969 and close to their R^2^ value. While the adjusted R^2^ value for TPC was 0.9953 and close to their R^2^ value. Both predicted R^2^ values were in reasonable agreement, where the difference was less than 0.2^22^. The lack of fit was not statistically significant at p > 0.05, showing a high degree of fit with the model for predicting the extraction yield^27,46^. The developed models were satisfactory to describe the connection between the independent and the response variables.

**Table 3:** Analysis of variance of the predicted models for quercitrin and TPC from *C. caudatus* leaves.

	Y_1_		Y_2_	
	Coefficient	p-value	Coefficient	*p*-value
β_0_	151.264		41.933	
**Linear**		0.000*		0.000*
X_1_	− 0.964	0.000*	− 0.582	0.000*
X_2_	0.085	0.852	0.140	0.063
X_3_	− 9.135	0.005*	3.871	0.148
**Quadratic**		0.000*		0.001*
X_1_^2^	− 0.062	0.000*	0.094	0.001*
X_2_^2^	− 6.932	0.000*	− 7.204 × 10–5	0.001*
X_3_^2^	0.153	0.003*	− 0.054	0.337
**Interaction**		0.003*		0.089
X_1_X_2_	− 2.542 × 10^–4^	0.263	1.254 × 10–4	0.747
X_1_X_3_	0.1053	0.001*	− 0.008	0.749
X_2_X_3_	3.419	0.435	− 0.002	0.020*
R^2^	99.30		99.62	
Adj. R^2^	89.69		95.53	
Pre. R^2^	98.04		98.95	
Lack of fit	0.135		0.413	

Parameter values with * is significant term at *p* ≤ 0.05.

The positive and negative sign of the coefficient shown is related to effect of increase or decrease in its value. In the case of quercitrin, the SLR and time had the most significant effect in the linear term, both showing a negative coefficient value, while particle size showed a less significant effect with a positive coefficient sign. The quadratic term between three variables had a greater effect to influence the quercitrin yield, whereas the interactive effect was only significant with a negative sign between the SLR and extraction time. This implies that the higher yield of quercitrin could be achieved when reducing the SLR and extraction time. After considering an only significant effect, the final regression model of quercitrin was given in Eq. (3):

3 $$\begin{aligned} {\text{QR content }}\left( {Y_{1} } \right) \, & = { 151}.{264 } - \, 0.{\text{964X}}_{{1}} {-}{9}.{\text{135X}}_{{3}} {-} \, 0.0{\text{62X}}_{{1}}^{{2}} {-}{ 6}.{932} \times {1}0^{{ - {5}}} {\text{X}}_{{2}}^{{2}} + \, 0.{\text{153X}}_{{3}}^{{2}} \\ & \quad + \, 0.{1}0{\text{5 X}}_{{1}} {\text{X}}_{{3}} . \\ \end{aligned}$$

For the TPC, among the independent variables, only the SLR displayed a strong significant linear effect with a negative coefficient sign. The quadratic term was significant and corresponded to the SSR and particle size, except for the extraction time. The interactive effect of particle and time were significant and had a greater effect on the TPC. The negative coefficient denoted that decreasing the particle size and extraction time to a certain level could result in a higher TPC while applying a higher SLR could increase the yield. As for the final equation for prediction of TPC, after excluding the insignificant terms, the final equation was given in the following Eq. (4):

4 $${\text{TPC}}\left( {{\text{Y}}_{{2}} } \right) \, = { 41}.{933 } - \, 0.{\text{582X}}_{{1}} + \, 0.0{\text{94 X}}_{{1}}^{{2}} {-}{ 7}.{2}0{4 } \times {1}0^{{ - {5}}} {\text{X}}_{{2}}^{{2}} {-} \, 0.00{\text{2 X}}_{{2}} {\text{X}}_{{3}} .$$

### Analysis of the contour and surface plot

**Figure 1:**
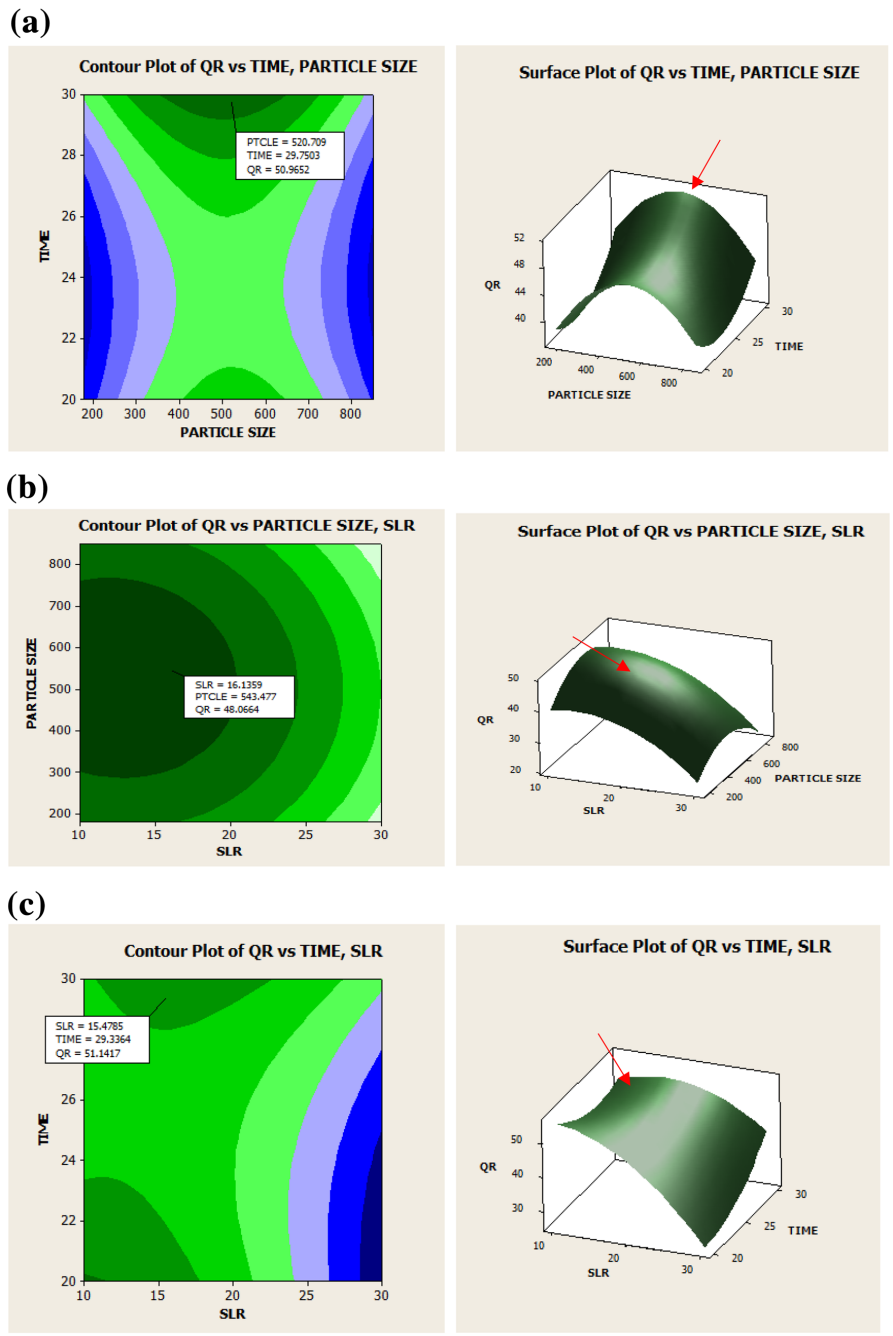
2D contour plot (left) and 3D surface plot (right) showing the influence of time and particle size (**a**), time and SLR (**b**) and particle size and SLR (**c**) on quercitrin content. The line on surface plot demonstrating the predicted optimal point.

**Figure 2:**
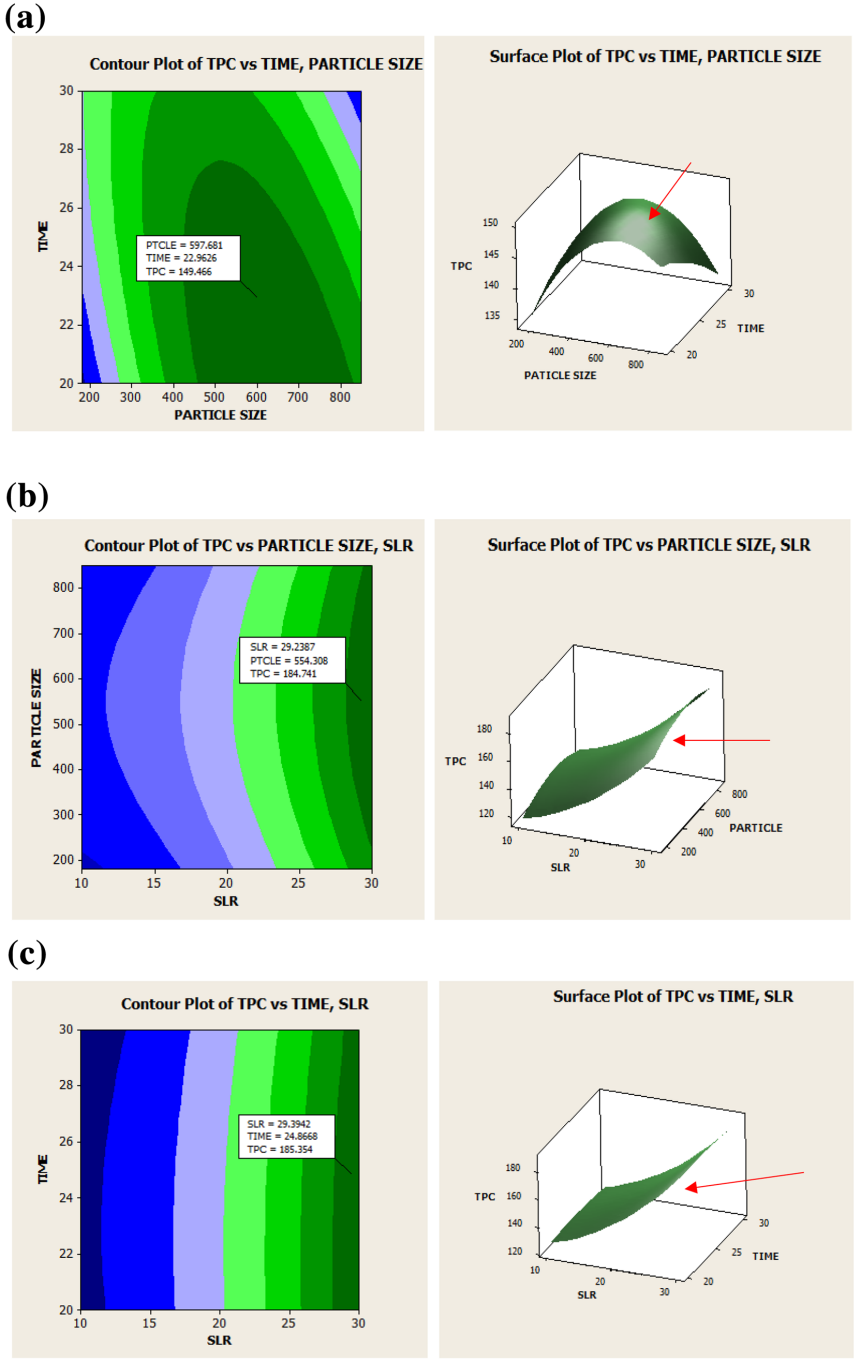
2D contour plot (left) and 3D surface plot (right) showing the influence of time and particle size (**a**), time and SLR (**b**) and particle size and SLR (**c**), on TPC. The line on the surface plot demonstrating the predicted optimal point.

The two-dimensional (2D) contour and three-dimensional (3D) surface plots are used to graphically represent the interactive effects of the studied variables to the responses^47^. The response plots of quercitrin content are presented in Fig. 1a–c, while Fig. 2a,b shows the response plots of TPC. Figure 1a,b displays the impact of particle size and time on quercitrin and TPC extraction while holding SLR at 1:20 g/mL. In decreasing the particle size from 850 to 500 µm and by increasing the time 25 min shows increment yields in quercitrin and TPC. We had also examined these effects in our preliminary screening, which larger particle size of 2500 µm recovered less quercitrin and TPC compared to the smaller particle below 850 µm (see Supplementary Information). According to Mohd^39^, the maximum concentration of quercitrin from the *Melastoma malabthricum* leaf could be extracted with an average particle size of 450 µm, using an ultrasonic extraction. In general, the extraction yield improved when reducing the particle size from crushed to powdered particle^39,48,49^. When the particle size is reduced, extraction yield increases due to a higher contact surface area of the sample with the solvent. In addition, the reduction of the particle size allows for a better mass transfer process due to the reduction in the diffusion path and the enlargement of the contact surface area^50^. Vuong and his team^51^ inspected the effect of particle size (< 1 mm) of green tea on the extraction efficiency. At the respective conditions, small particles sink to the bottom of the extraction vessel, and additional steps for the extraction are needed (e.g., separation, filtration, or centrifugation) to clear the sediment. This may reduce the extraction efficiency as the steps required cost, energy, and time^47^.

Figures 1b and 2b shows the mutual interaction of particle size and SLR on quercitrin and TPC extraction with the time set at 25 min. For the SLR, the quercitrin yield was slightly increased with increasing SLR (1:10 g/mL to 1:20 g/mL) and reducing particle size (850–500 µm). As shown in Fig. 1b, the maximal content of quercitrin reaches 48.07 mg/g dw using SLR at 16.14 g/mL and particle size at 543 µm. In Fig. 2b, the TPC value could be increased when SLR higher than 1:30 g/mL was used for the extraction (184.74 mg GAE/g dw). The results are consistent as reported by Ma et al.^40^, Mohd^39^ and Chen et al.^52^. With the increase in SLR, viscosity of the medium solution decreases, and increases in concentration gradient leading to more solvent access into the material and producing a higher extraction yield.

The present study had examined the mutual interaction of ultrasonic extraction time and SLR on quercitrin and TPC extraction. The particle size was set constant at 515 µm. As displayed in Figs. 1c and 2c, the extraction was favoured if a longer time was used. A longer time increase up to 30 min gradually increased both extraction yields and solute release as it promoted the extension, cell wall degradation. The increase in sonication time also caused the power and solvent temperature to increase, thereby effecting the extraction yield^53,54^. However, in some cases, extraction efficiency may decrease due to the prolonged sonication time as it reduces the permeability of the solvents to the cell walls due to the over-suspended impurities and affecting its mass transfer^31,39^. In the present study, the solvent temperature was controlled at 50 °C to avoid structural damage to the solute and to increase the quercitrin and TPC yields. Mohd^39^ reported ultrasonic extraction time of 15 min could be optimum for quercitrin from *M. malabathricum* leaves. However, the results were found to be contradicted with Zulkiply^35^ who observed ultrasonic extraction time of 300 min is the best to achieve the maximal TPC yield from *C. caudatus* leaves extract.

### Predicted optimal extraction conditions and verification

The optimal conditions of the studied variables were determined by setting the goal at the maximum responses using the optimizer plot. The overall desirability of the regression models was achieved at 0.9508, which is close to 1. The predictive maximum values of quercitrin and TPC were 44.26 mg/g dw, and 175.64 mg GAE/g dw, respectively under the optimised condition of SLR 1:28 (g/mL), particle size 485 µm, and extraction time 30 min (Table 4). Meanwhile, the values from the actual experimental work were recorded at 42.57 mg/g dw and 169.75 mg GAE/g dw, respectively. These measured values were consistent and close to the values predicted by the model equation. The percentage error between the predicted and experimental values was less than 4%, which justifies an appropriate suitability. Through this validation study, the above-developed regression models were found to be suitable to optimise the ultrasonication process.

**Table 4:** Quercitrin and TPC yield under optimal ultrasonic extraction conditions.

Responses	Experimental	Predicted	% Error
Quercitrin (mg/g dw)	42.57	44.26	3.81
Total TPC (mg GAE/g dw)	169.75	175.64	3.72

### Comparison of ultrasonic extraction with Soxhlet

Extraction by ultrasonics is a novel extraction method with advantages such as higher extraction efficiency, lower solvent consumption, and shorter extraction time^31,55^. Many studies have been carried out in the literature to compare the influence of extraction methods on extraction yields, such as bioactive compounds, phenolic compounds, and antioxidant properties of various plant species^56,57^. Specifically, extraction via ultrasonic and microwave techniques has proved to be the most effective extraction method for a higher recovery of bioactive compounds from *Panax ginseng* and *Gingko biloba*^56^.

Quercitrin, TPC, and antioxidant property extracted using ultrasonics were found to be higher than Soxhlet extraction (Table 5). This proved that ultrasonic extraction has successfully enhanced the extraction yields from the plant extract. Seyedreihani et al.^16^, revealed that the TPC in the *C. caudatus* aqueous extract was 37.41 mg GAE/g dw. TPC in the 80% ethanolic extract was 15.8 mg GAE/100 g dw^13^. Comparatively, the low value of TPC is due to the authors not performing optimization of the extraction parameters in their study. It is reported that the beneficial active compounds from the plant are mostly from the flavonoids derivatives^9,16^. Adarwulan et al.^58^ reported that the sum flavonoids compounds (consisting of quercetin, kaempferol, luteolin, apigenin) on a dry basis was 3.72 mg/100 g. Compared with our result, quercitrin which is a major flavonoids constituent in the plant extract was 11-fold higher than the reported sum flavonoids compound. Sharifuldin et al.^14^ had reported the average value of quercitrin in the 75% ethanolic extract using Soxhlet ranged from 8.13 to 11% (w/w, in dw). Yusoff et al.^9^ had reported the concentration of quercitrin in the methanol was about 29.66 mg/g dw. While, in the study by Seyedreihani et al.^16^, the water extract contains 36.90 mg/g of quercitrin. The values reported of this major compound among the *C. caudatus* extracts differ due to several reasons such as maturity, treatment or environmental changes^59^. According to Mediani et al.^13^, quercitrin showed the highest concentration (9.58 mg/g) in the dry season and the 8th-week development stage. In a previous study by Mohd Khairi et al.^32^, the effect of different extraction methods on ascorbic acid content was compared between ultrasonic, maceration, and Soxhlet^32^. Their established results showed that the ultrasonic extraction method was most effective with 26.59 mg/g dw ascorbic acid obtained from the plant extract. The antioxidant inhibition based on the IC_50_ of the extract in ultrasonic extraction and Soxhlet were 20.83 ± 1.33 mg/L and 36.80 ± 0.68 mg/L, respectively. The results notably increased 0.57-fold compared to the results produced by the Soxhlet method. Interestingly, this value is also close to the IC_50_ of the standard ascorbic acid value at 13.25 ± 0.45 mg/L. In summary, the results demonstrated that the extraction method plays an important role in recovering extraction yield.

**Table 5:** Comparison of extraction yield between ultrasonic and Soxhlet extraction methods.

Method	Extraction yield		
	Quercitrin (mg/g dw)	TPC (mg/g dw)	IC_50_ (mg/L)
Soxhlet	35.50 ± 0.24	125.97 ± 1.64	36.80 ± 0.68
Ultrasonic	42.57 ± 0.51	169.75 ± 0.63	20.83 ± 1.33

Values are presented as mean ± SD (n = 3).

Ultrasonic extraction was found to be a robust method for high yield of quercitrin, TPC, and antioxidant property from *C. caudatus* leaves extract. Nevertheless, the reduction in both extraction time and required solvent makes ultrasonic extraction more efficient than the Soxhlet method. The difference in results could be associated with the process of extraction involved.

### Evaluation of the antimicrobial potential of* C. caudatus* extract

**Figure 3:**
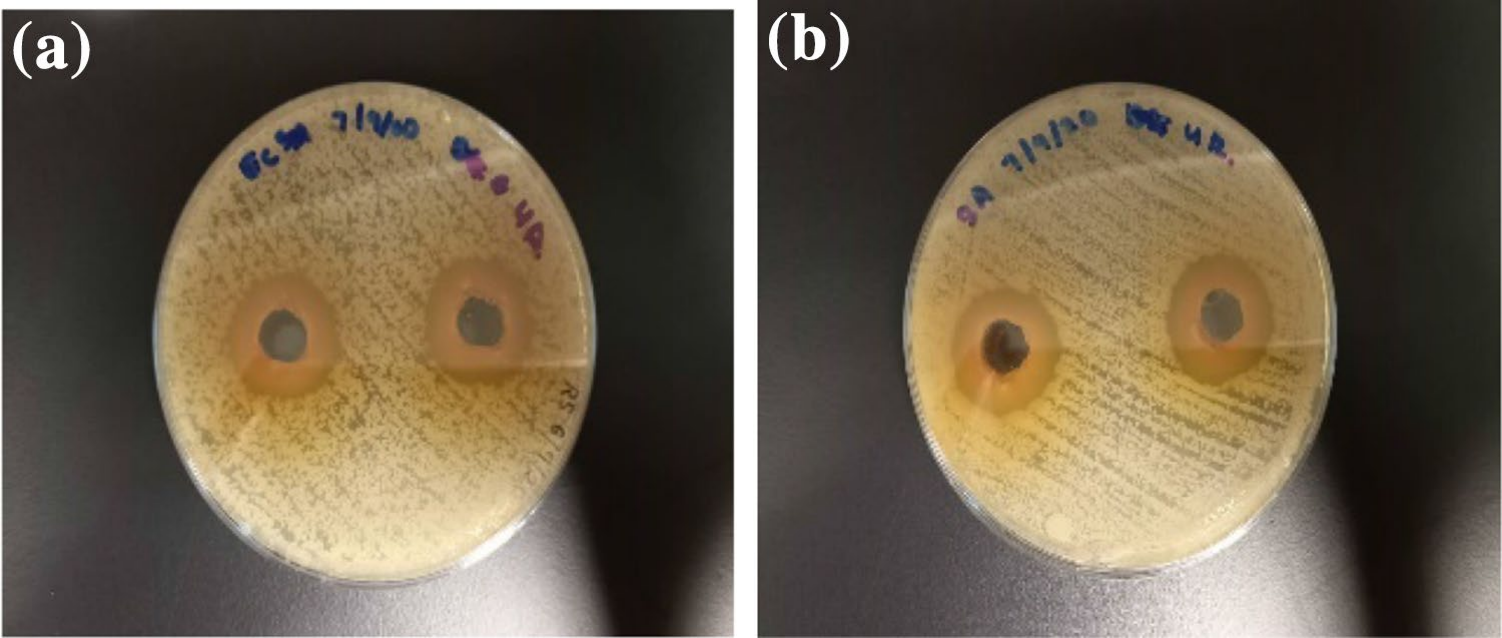
Inhibition zone of *C. caudatus* extract on (**a**) *E. coli*, (**b**) *S. aureus.*

The antimicrobial potential of *C. caudatus* extract was determined against two human pathogenic bacteria, namely, *Staphylococcus aureus* (*S. aureus*) and *Escherichia coli* (*E. coli*). Results of the inhibition growth of these Gram-positive and Gram-negative bacteria present their apparent clear zone as shown in Table 6. After incubation at 37 °C for 24 h, the inhibition growth of *S. aureus* had produced a clear zone of diameter 22.67 ± 0.57 mm, while *E. coli* had produced a clear zone of diameter 21.57 ± 0.50 mm (Fig. 3). The inhibition zone of quercitrin was similar to the positive control, forming a clear zone of diameter 23.31 ± 0.72 mm for *S. aureus*, and 22.86 ± 0.31 mm for *E. coli* at the tested concentration (1 mg/mL). The finding proved that quercitrin, as an active plant compound in *C. caudatus* 80% ethanolic extract, is responsible for antimicrobial activity. A similar result was reported by Yusuff et al.^9^ who found the antimicrobial activity of *C. caudatus* leaves extract might be due to the presence of quercitrin. Besides, quercetin and its derivatives produced an extraordinary effect of bacterial resistance against several human pathogenic bacteria such as *Staphylococcus aureus*, *Staphylococcus epidermidis*, *Porphyromonas gingivalis, Bacillus subtilis, and Escherichia coli*^60–62^. The results reveal the capabilities of the extract as an anti-microbial agent, similar to the previous studies^7,8^.

**Table 6:** Antimicrobial activity of *C. caudatus* leaves extract.

Sample	Zone of inhibition (mm)	
	*Staphylococcus aureus*	*Escherichia coli*
*C.caudatus* extract	22.67 ± 0.57	21.57 ± 0.50
Quercitrin standard	23.31 ± 0.72	22.86 ± 0.31
Streptomycin	24.11 ± 0.12	24.11 ± 0.12

### Identification of compounds of bioactive compounds by HPLC

**Figure 4:**
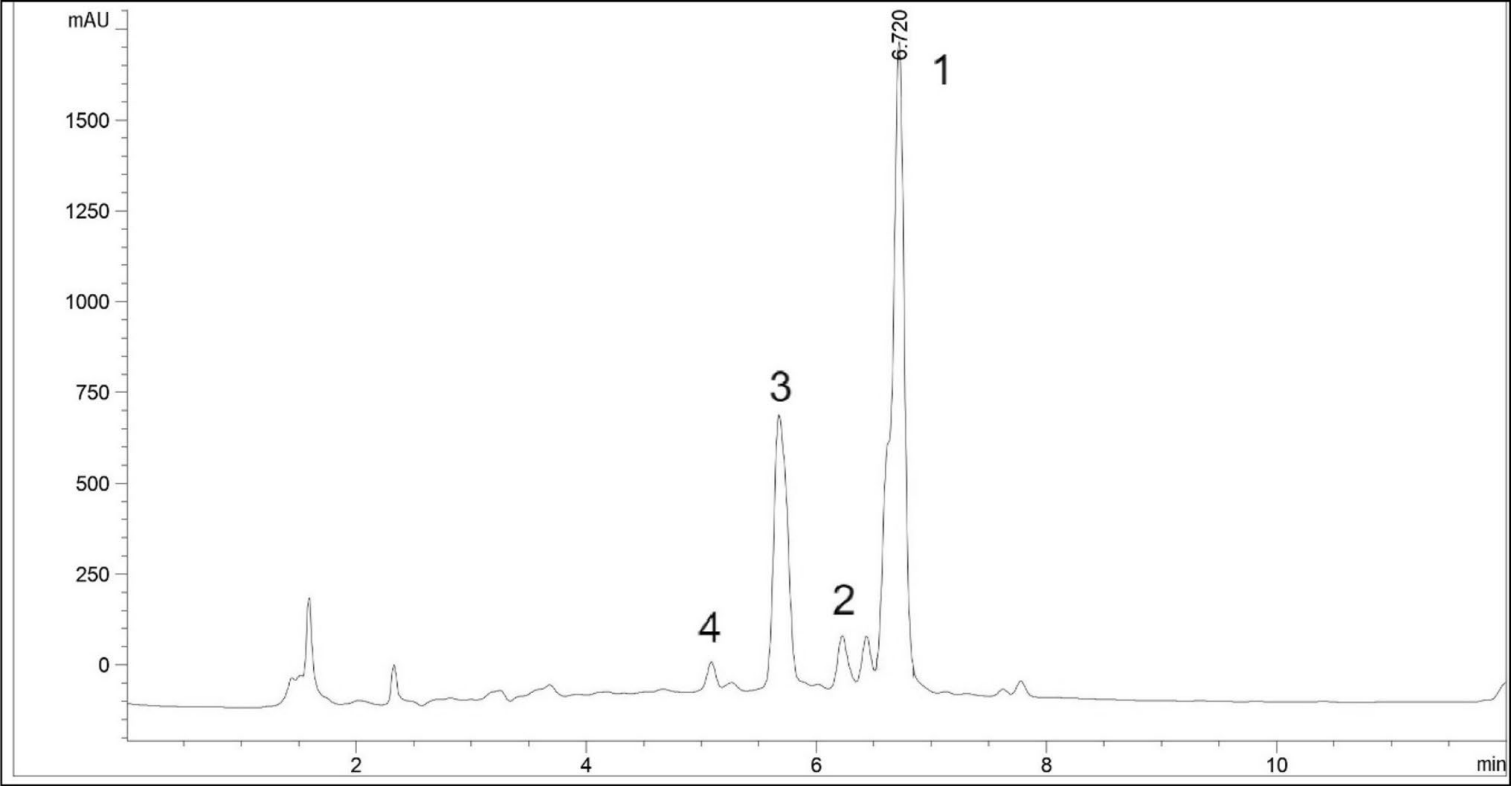
HPLC chromatogram of *C. caudatus* extract at 260 nm. The peak as follow: 1, quercitrin; 2 quercetin glycoside; 3, unknown peak; 4, rutin.

The chromatographic profile of the sample extract identified three compounds, namely, quercitrin, quercetin glycoside, and rutin (Fig. 4). The major compound was identified as quercitrin, which was eluted at 6.73 min. Quercetin glycoside and rutin were eluted at 6.34 min and 5.08 min, respectively. This HPLC chromatogram profile were similar to the chromatogram profiles of Mediani et al.^13^, Sharifuldin et al.^14^ and Seyedreihani et al. ^16^ in their sample extracts. However, the elution time of our compounds was much faster. The previous articles have reported that quercitrin was eluted at 19.93 min and 28.64 min^12,15^. The use of 0.3% formic acid and acetonitrile as mobile phases (and considered a suitable developed gradient system) has led to the rapid compound elution and is useful for the quantification of quercitrin in the sample extract. There was also one unidentified peak detected at 5.74 min. The unknown peak may possibly be another phenolic compound or other flavonoids. This compound was also important and needed to be further identified as its presence constitutes the second largest peak in the samples. Further work is recommended to identify this unknown compound by using a more sophisticated analytical technique, such as HPLC mass spectrometry.

## Conclusions

This study is perhaps the first to report on the use of the ultrasonic extraction method for the extraction and separation of quercitrin and TPC from *C. caudatus* leaves. According to the results, the optimum yield of quercitrin and TPC were 42.57 mg/g and 169.75 mg GAE/g respectively. The optimum extraction variables involved using a SLR of 1:28 (g/mL), particle size of 485 µm and extraction time of 30 min. The ultrasonic extraction method has been proven to increase the yield of the extracted components and to have additional advantages such as shorter time, lower solvent amount, and low operational cost over the Soxhlet extraction method. The results demonstrated the capabilities of the extract as an antimicrobial agent. This developed ultrasonic extraction method can contribute to the development of extraction protocols, or for further fractionation of bioactive compounds from *C. caudatus* leaves or other medicinal plants.

## Supplementary Information

Supplementary Figure 1.

## Data Availability

The datasets generated or analysed during this study are included in this article.
