# Supplementary material for: Enhancing recovery of bioactive compounds from Cosmos caudatus leaves via ultrasonic extraction
Source: Sci Rep. 2021 Aug 27;11:17297. doi: 10.1038/s41598-021-96623-x (PMC8397774; doi:10.1038/s41598-021-96623-x)
Supplement: Supplementary file 1 — Supplementary Figure 1. [file 41598_2021_96623_MOESM1_ESM.pdf]

Enhancing Recovery of Bioactive compounds from *Cosmos caudatus* leaves via Ultrasonic Extraction

Norliza Abdul Latiff[1,3*], Pei Ying Ong[3], Luqman Chuah Abdullah[1*], Siti Nor Azlina Abd Rashid[3], Noor Akhmazillah Mohd Fauzi[4] and Nor Amaiza Mohd Amin[2]

[1]*Department of Chemical and Environmental Engineering, Faculty of Engineering, Universiti Putra Malaysia, Serdang, 43400, Selangor, Malaysia*

[2]*Department of Process and Food Engineering, Faculty of Engineering, Universiti Putra Malaysia, Serdang, 43400, Selangor, Malaysia*

[3]*Innovation Centre in Agritechnology, Universiti Teknologi Malaysia, Muar, 84600, Johor, Malaysia*

[4]*Department of Chemical Engineering Technology, Faculty of Engineering Technology, Universiti Tun Hussein Onn Malaysia, Pagoh Higher Education Hub, Muar, 84600, Johor, Malaysia*

Correspondence Author: L.C. Abdullah & N. A. Latiff.

Email : chuah@upm.edu.my Tel. +60-3-86567120; norlizaabdlatiff@utm.my Tel. +60-6-9742856

Running title: Data obtained from preliminary experiment to determine the extraction parameters and analysed its effect on the yield of quercitrin and TPC.

Supplementary Figures

A

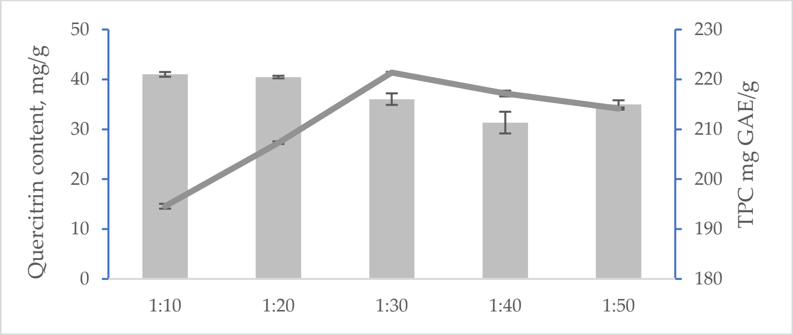

B

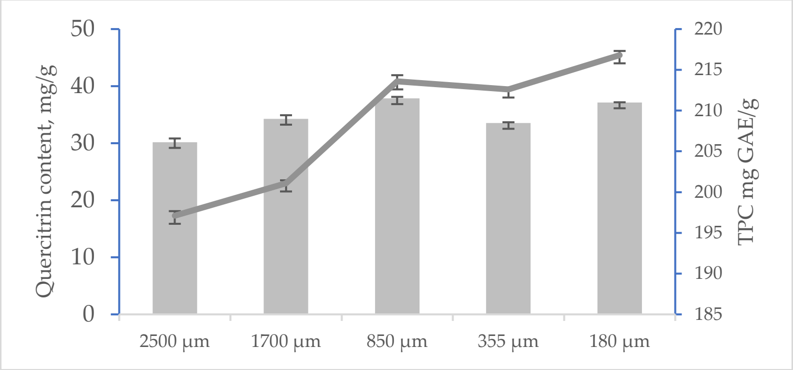

C

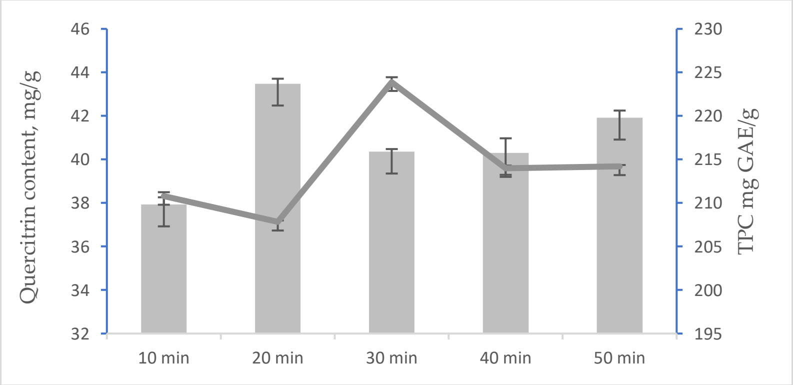

D

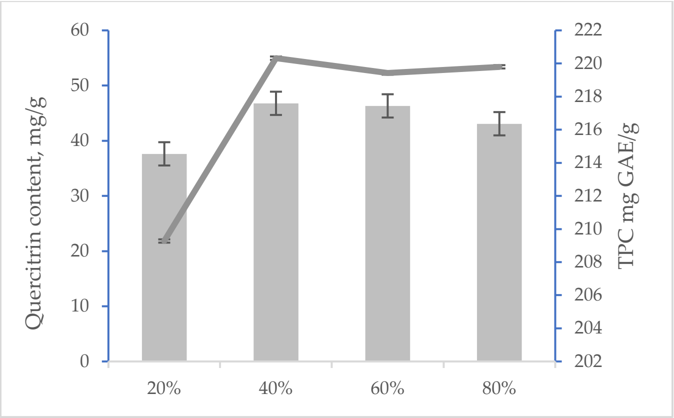

Figure 1S. Effects of solid to liquid ratio, SLR (**A**, extracted at 80% ethanol, Particle size 850 µm, 30 min, 40% amplitude), Particle size (**B**, extracted at 80% ethanol, SLR 1:20 g/mL, 30 min, 40% amplitude), Time (**C**, extracted at 80% ethanol, SLR 1:20 g/mL, Particle size 355 µm, 30 min, 40% amplitude) and Amplitude (**D**, extracted at SLR 1:20 g/mL, Particle size 355 µm, 30 min) on yield of quercitrin (bar graph) and TPC (line graph), by single factor experiment. A total 19 test with three replication each were performed.
